# Supplementary material for: Genetic determination and JARID2 over-expression in a thermal incubation experiment in Casque-Headed Lizard
Source: PLoS One. 2022 Jul 7;17(7):e0263804. doi: 10.1371/journal.pone.0263804 (PMC9262179; doi:10.1371/journal.pone.0263804)
Supplement: Attachment — Submitted filename: Suarez-Varon.et.al.Rebuttal_Letter.pdf [file pone.0263804.s004.pdf]

Diego Cortez, PhD
Centro de Ciencias Genómicas
UNAM
Ave. Universidad s/n. CP-62210. Cuernavaca. México
email: dcortez@ccg.unam.mx

Oswaldo Hernández, PhD
Laboratorio de Herpetología
Universidad Autónoma del Estado de México
Instituto Literario #100 Centro. CP-50000. Toluca. México
email: ohg@uaemex.mx

10 March 2022

Patrick R Stephens, Ph.D.
Academic Editor
PLOS ONE

Dear Dr. Stephens,

The authors would like to thank you and the reviewers for their thoughtful insights into our manuscript. These comments and suggestions have largely improved the quality of our work. We have addressed all of the comments in the manuscript (of Reviewer#1 and edits of J. Walker). A detailed list of the corrections is shown below (comments of Reviewer#1). The newly revised version of the manuscript includes the editor and reviewers' comments. Throughout the entire manuscript, several paragraphs were rewritten for better understanding, and the discussion was expanded.

Academic Editor's comments:

Both reviewers were enthusiastic about the topic and the potential importance of the findings. However, both authors found issues with the text. James Walker volunteered to be identified and made a number of editorial suggestions. You can find a document in the PLOS One editorial system with his suggest edits (the final version that he sent to me is labeled JWalker edits). These are only meant to be taken as suggestions, and I leave it to your discretion which of them you wish to implement. However, many of his suggestions seem sound to me so please do carefully consider his feedback.

The other reviewer identified more substantial issues, particularly areas where the clarity of the manuscript could be improved. Please do respond directly to each of the issues raised by this reviewer in your rebuttal letter.

Answer:

We thank the Academic Editor for these comments. We have now carefully studied the suggestions made by Dr. Walker. We have accepted most of these suggestions as they clearly improved the text. We have also clarified the questions raised by Dr. Walker regarding the use of some words, the meaning of some sentences, and the common names or families of reptiles we described. Additionally, we have addressed the comments raised by Reviewer#1 regarding the lack of important information in the Methods and the

Discussion. Please find below our point-by-point answer to Reviewer#1 comments. We addressed Dr. Walker suggestion's in the marked-up copy of your manuscript.

Reviewer#1's comments:

1) Reviewer#1:

Due to the small sample sizes and limited temperature range the available eggs could be incubated at, this study was unable to definitively conclude whether or not the species has sex reversal. As such, I strongly suggest that the title be changed to more accurately reflect the findings of the study. It currently reads like sex reversal was shown to occur in this species, and the short title "Lack of sex reversal in casque-headed lizards" is actually what was found.

Answer:

We thank Reviewer#1 for the detailed revision of our work. Following her/his advice, we have changed the title of the study to reflect the main findings. The new title is: "Genetic determination and JARID2 over-expression in a thermal incubation experiment in Casque Headed Lizard". **Page 1** in the revised version of the manuscript.

2) Reviewer#1:

I noticed that 82 eggs were incubated, but only 48 across the three incubation temperatures reached the target developmental stages. That seems like quite a high mortality rate - are the authors able to comment on this? Was the mortality observed at a particular temperature, were perhaps some of the eggs not actually viable when they were initially incubated? The temperatures aren't particularly extreme, so I would be surprised to see temperature specific mortality. If sex specific mortality has occurred this could affect the results presented in the manuscript.

Answer:

We thank Reviewer#1 for this comment. We agree that the number of eggs that were incubated and the number of eggs reported for the histological/genotypic analyses do not match. We have now corrected this mistake. We would like to clarify that of the 22 females, a total of 130 eggs were obtained. These eggs were randomly assigned to three incubation temperatures (26, 29 and 32°C). Of these, 42 eggs (32%) became contaminated with fungal infection and the embryos died with no effect of temperature on mortality rates ($X^2 > 0.05$). Of the 88 eggs that successfully reached the target developmental stages, 40 were used in a parallel study that aimed to establish the effect of incubation temperatures (26, 29 and 32°C) on the development of the embryos: Suárez-Varón. etal. (2021). REVISTA MEXICANA DE BIODIVERSIDAD. 92:923795 (http://rev.mex.biodivers.unam.mx/index.php/es/variacion-del-estadio-embrionario/). Thus, we used 48 eggs to study the association between incubation temperature, genotype, and the histology of gonads (present study). We have added more information to the Methods. **Page 5, first paragraph**.

3) Reviewer#1:

My major concern with the manuscript is the tissue used for the RNA-seq analysis. As the methods are currently written I am not entirely clear what tissue was used. Under the "laboratory conditions" section of the methods the posterior part of the embryo was used for histology, so was clearly the part of the embryo containing the gonads. Then the rest of the embryo was preserved for further genetic analysis. This is quite a large part of the embryo containing a wide variety of different tissue types. Then in the "RNA extraction and RNA-seq analysis" section samples were obtained from whole embryos. Were they sub sampled from the other half of the embryo, or were these whole embryos that weren't also used for histology? Regardless, what was the actual tissue type that was sequenced? Or was a large part of the embryo homogenised?

Answer:

We thank Reviewer#1 for this comment. We agree that the Methods are not clear about the tissues used for the analyses. Embryos that reached the target developmental stages were divided into two segments. An upper segment that comprised the head and eyes. And a lower segment where the gonads were located. Each individual was assigned a number so we could match the genetic material versus the histology of the gonads. The upper (head/eyes) segment was then divided longitudinally into two fragments of equal size. One that was homogenized and from which we extracted DNA for the genotypic verification of the Y chromosome, and a second fragment that was also homogenized and from which we attempted to purify RNA; RNA was degraded in many samples though. We have added more information to the Methods. **Page 5, first and last paragraphs; Page 6, second paragraph**.

4) Reviewer#1 comment:

This information is important not only for the methodological clarity of this manuscript, but also because the RNA-seq data has been deposited publicly on SRA, so any other researchers looking to use this data need to have a clear understanding of what the actual sample was. An additional concern is if the same tissue type wasnt used, this significantly affects the validity of the differential gene expression analysis. Based on the low number of differentially expressed genes, I assume that the tissues were consistent between samples, but this does need to be explictly stated.

Answer:

Reviewer#1 is correct. Using different tissues to conduct differential gene expression analysis may have led to potentially odd results. Besides, detailed information about the samples used is important to replicate our results. As explained above, we used half of the upper segment (head/eyew) of the embryos for DNA/RNA extractions. Tissues were homogenized prior to the purification of the genetic material. We have added more information to the Methods. **Page 5, first and last paragraphs; Page 6, second paragraph**.

5) Reviewer#1 comment:

The discussion feels a little unfinished, and would benefit from a short concluding paragraph, particularly highlighting future research directions, or how it might be possible to definivitely show whether or not the species has sex reversal.

Answer:

We thank Reviewer#1 for this comment. We have added a new paragraph at the end of the Discussion where we describe the limitations of the work and future research directions. **Page 12, second paragraph**.

Please do not hesitate to contact us if you need any further information or clarification.

With best wishes,

Dr. Diego Cortez

Dr. Oswaldo Hernández
