# Supplementary material for: Genetic determination and JARID2 over-expression in a thermal incubation experiment in Casque-Headed Lizard
Source: PLoS One. 2022 Jul 7;17(7):e0263804. doi: 10.1371/journal.pone.0263804 (PMC9262179; doi:10.1371/journal.pone.0263804)
Supplement: Attachment — Submitted filename: Suarez-Varon.et.al.Response_to_Reviewers.docx [file pone.0263804.s005.docx]

Diego Cortez, PhD

Centro de Ciencias Genómicas

UNAM

Ave. Universidad s/n. CP-62210. Cuernavaca. México

email: dcortez@ccg.unam.mx

Oswaldo Hernández, PhD

Laboratorio de Herpetología

Universidad Autónoma del Estado de México

Instituto Literario #100 Centro. CP-50000. Toluca. México

email: ohg@uaemex.mx

14 June 2022

Patrick R Stephens, Ph.D.

Academic Editor

PLOS ONE

Dear Dr. Stephens,

The authors would like to thank you for your thoughtful insights into our manuscript. These suggestions have largely improved the quality of our research. We have addressed all the comments in the manuscript (clean copy version) and a detailed list of the adjustments made can be seen below (responses are in bold).

Academic Editor’s comments:

My apologies for how long this decision took. Since we were unable to obtain a second review in a timely fashion I have acted as the second reviewer for this revision. I think there are a few minor revisions you still need to make, but overall this is a really solid work. Once you make a few small changes to enhance readability, I believe this will be ready to go. Here are my additional suggestions:

Line 58:

Change “their sex determination mechanisms remain poorly understood”

To “the factors that drive variation in sex determination mechanisms among species remain poorly understood”

Your next sentence demonstrates that we know a lot about how sex determination works in squamates in general, making the first sentence seem like an exaggeration as written.

**Our answer: Done, manuscript was modified to attend this suggestion (line 58).**

Line 76:

Delete “however”

Should read:

. . .chromosomes, non-avian reptiles have . . .

**Our answer: Done, manuscript was modified to attend this suggestion (line 77).**

Methods:

PLOS is pretty flexible about formatting. However, for readability the formatting should be internally consistent. I suggest that you follow the formatting you use in the Results for headings and subheading throughout.

Line 112: bold the heading like in the discussion

**Our answer: Done, manuscript was modified to attend this suggestion (line 113).**

Line 121: there should be a line of space in between 121 and 122, bold the heading

**Our answer: Done, manuscript was modified to attend this suggestion (line 124).**

Line 141: bold the heading

**Our answer: Done, manuscript was modified to attend this suggestion (line 143).**

Line 148: bold the heading

**Our answer: Done, manuscript was modified to attend this suggestion (line 150).**

Line 165: bold the heading

**Our answer: Done, manuscript was modified to attend this suggestion (line 167).**

Line 261:

By “top-second gene” do you mean the gene with highest expression difference between 26°C and 32°C? If that is the case, I would delete the phrase “top-second” as I find it really unclear.

**Our answer: Done, we delete the phrase “top-second” (line 263).**

I’m also not sure what’s “remarkable” about this result from the intro. In this context “remarkably” implies a result running counter to what most people would expect or that is unusual or unexpected in some other way. However, from what we have read up to this point it’s not clear why you think the result is remarkable.

I would change this sentence to

“JARID2 was the gene with the highest expression difference between 26°C and 32°C . . .”

**Our answer: Done, manuscript was modified to attend this suggestion (line 262).**

In the discussion, where you immediately explain what’s remarkable about the result, I think it’s fine to use this phrasing.

**Our answer: Thanks.**

Line 322: I would delete “top-second” here as well. I find the phrase really unclear.

Change the sentence to:


“Remarkably, we observed that JARID2 was the gene with the highest expression difference between low and high incubation temperatures.”

**Our answer: Done, manuscript was modified to attend this suggestion (line 324).**

Please do not hesitate to contact us if you need any further information or clarification.

With best wishes,

Dr. Diego Cortez

Dr. Oswaldo Hernández
